# High Frequency of Endothelial Colony Forming Cells Marks a Non-Active Myeloproliferative Neoplasm with High Risk of Splanchnic Vein Thrombosis

**DOI:** 10.1371/journal.pone.0015277

**Published:** 2010-12-09

**Authors:** Vittorio Rosti, Elisa Bonetti, Gaetano Bergamaschi, Rita Campanelli, Paola Guglielmelli, Marcello Maestri, Umberto Magrini, Margherita Massa, Carmine Tinelli, Gianluca Viarengo, Laura Villani, Massimo Primignani, Alessandro M. Vannucchi, Francesco Frassoni, Giovanni Barosi, on behalf of the AGIMM Investigators

**Affiliations:** 1 Unit of Clinical Epidemiology and Center for the Study of Myelofibrosis, IRCCS Policlinico S. Matteo Foundation, Pavia, Italy; 2 Unit of Clinica Medica 1, Department of Internal Medicine, IRCCS Policlinico S. Matteo Foundation, Pavia, Italy; 3 Unit of Hematology, Department of Critical Care, University of Florence and Istituto Toscano Tumori, Florence, Italy; 4 Department of Surgery, IRCCS Policlinico S. Matteo Foundation, Pavia, Italy; 5 Laboratory of Biotechnology, IRCCS Policlinico S. Matteo Foundation, Pavia, Italy; 6 Biometric Unit, Fondazione IRCCS Policlinico S. Matteo Foundation, Pavia, Italy; 7 Unit of Clinical Immunology, Immunohematology, and Transfusion Service, IRCCS Policlinico S. Matteo Foundation, Pavia, Italy; 8 Gastroenterology 3 Unit, IRCCS Cà Granda Ospedale Maggiore Policlinico Foundation, Milano, Italy; 9 Centro Cellule Staminali e Terapia Cellulare, Ospedale San Martino, Genova, Italy; Health Canada, Canada

## Abstract

Increased mobilization of circulating endothelial progenitor cells may represent a new biological hallmark of myeloproliferative neoplasms. We measured circulating endothelial colony forming cells (ECFCs) in 106 patients with primary myelofibrosis, fibrotic stage, 49 with prefibrotic myelofibrosis, 59 with essential thrombocythemia or polycythemia vera, and 43 normal controls. Levels of ECFC frequency for patient's characteristics were estimated by using logistic regression in univariate and multivariate setting. The sensitivity, specificity, likelihood ratios, and positive predictive value of increased ECFC frequency were calculated for the significantly associated characteristics. Increased frequency of ECFCs resulted independently associated with history of splanchnic vein thrombosis (adjusted odds ratio = 6.61, 95% CI = 2.54–17.16), and a summary measure of non-active disease, i.e. hemoglobin of 13.8 g/dL or lower, white blood cells count of 7.8×10^9^/L or lower, and platelet count of 400×10^9^/L or lower (adjusted odds ratio = 4.43, 95% CI = 1.45–13.49) Thirteen patients with splanchnic vein thrombosis non associated with myeloproliferative neoplasms were recruited as controls. We excluded a causal role of splanchnic vein thrombosis in ECFCs increase, since no control had elevated ECFCs. We concluded that increased frequency of ECFCs represents the biological hallmark of a non-active myeloproliferative neoplasm with high risk of splanchnic vein thrombosis. The recognition of this disease category copes with the phenotypic mimicry of myeloproliferative neoplasms. Due to inherent performance limitations of ECFCs assay, there is an urgent need to arrive to an acceptable standardization of ECFC assessment.

## Introduction

The classical Philadelphia negative (Ph-neg) myeloproliferative neoplasms, i.e. polycythemia vera, essential thrombocythemia, and primary myelofibrosis, consist of clonal malignancies characterized by the proliferation of one or more myeloid lineages that originate at the level of stem cells. The discovery of recurrent molecular abnormalities, like *JAK2*V617F and *MPL*W515L/K mutations [1], has reinforced the Dameshek's vision that these disorders have a common pathogenetic mechanism and presumably belong to an unique disease process [2]. The difficulties in dissecting the continuum of clinical phenotypes are reflected in the recently revised WHO classification [3], which arranges hematological, morphological and molecular parameters to separate the three clinical disorders. Even though this classification has endorsed the concept of “prefibrotic-early stage of primary myelofibrosis”, and “pre-polycythemic phase” of polycythemia vera to capture the stepwise evolution of the diseases, the overlapping of clinical phenotypes persists, and the category of “unclassifiable myeloproliferative neoplasms” continues to challenge the system [3]. Since a precise classification predicts different natural history, prognosis, and needs of therapy, to date, many studies have focused on discovering new biological markers that could help unraveling the phenotypic mimicry [4]–[8].

Recently, high mobilization of putative endothelial progenitor cells has been proposed as a new biological hallmark of myeloproliferative neoplasms. Colony forming unit-endothelial or CD34-, CD133-, vascular endothelial growth factor receptor 2-positive putative endothelial progenitor cells, have been found selectively increased in the blood of patients with myelofibrosis, specially in the early phase of the disease [9]–[11]. These findings have originated the hypothesis that endothelial progenitor cell-mediated neoangiogenesis could intervene in determining the phenotypic profile of myeloproliferative neoplasms. However, recent knowledge on the origin of endothelial progenitor cells have weakened this interpretation. Functional and phenotypic overlap between hematopoietic and endothelial progenitors have been documented, while monocyte lineage has been proven able to differentiate into endothelial cells in vitro [12], [13].

Advancement in endothelial progenitor cell identification and assessment has occurred in 2004 when Ingram et al. [14] described a new category of cells, namely endothelial colony forming cells (ECFCs), that isolated under precise culture conditions, are organized in a hierarchy of precursor stages with different and robust proliferative potential, and with vessel forming capacity in vivo. Therefore, ECFCs are now considered a distinct colony type originating from endothelial lineage [15].

In this paper we reconsider the clinical significance of endothelial progenitor cells in classical Ph-neg myeloproliferative neoplasms by applying the ECFC measurement to a large cohort of patients. We compared the number of these cells in the different categories of patients, and we tried to find correlations between the cells numbers and disease's characteristics.

## Materials and Methods

### Study overview

Since the ECFCs measurement suffers of the limitation that only a very low number of colonies are detectable in humans, we first designed a study that would adequately estimate within-subject variability in ECFCs measurement. With this purpose, thirty-one healthy volunteers (15 men and 16 women), and 29 subjects with myeloproliferative neoplasms were asked to be tested in two different occasions, with a maximum of 5 days in between.

The main body of the study consists in a cross-sectional analysis on 214 patients with classical Ph-neg myeloproliferative neoplasms. The study sample consisted of patients referred to the Center for the Study of Myelofibrosis of the Fondazione IRCCS Policlinico S. Matteo of Pavia, Italy, from March 2007 to March 2010.

Since during the study it became evident an association between high frequency of circulating ECFCs and history of splanchnic vein thrombosis, we collected samples from 13 additional patients with splanchnic vein thrombosis in whom the diagnosis of myeloproliferative neoplasm was thoroughly searched and excluded, with the aim of investigating the causal relationship between splanchnic vein thrombosis and ECFCs mobilization. Samples were obtained from patients referred to the Gastroenterology Unit of the Maggiore Hospital Foundation of Milan or from Division of Hematology of the Careggi Hospital of Florence.

The policies for collection and use of blood samples were approved by the institutional review board of the IRCCS Policlinico S. Matteo Foundation, and all patients gave written consent for the donation of samples.

### Study Cohorts

For the cross-sectional analysis, we selected patients for being at diagnosis of disease or after diagnosis but without being receiving any disease-modifying therapy. All patients had their initial diagnosis re-evaluated by reviewing the diagnostic bone marrow biopsy by an experienced pathologist (UM). Fifty nine patients were diagnosed with polycythemia vera (n = 38) or essential thrombocythemia (n = 21) according to the WHO criteria [3]. One hundred and six patients were diagnosed with primary myelofibrosis according the WHO criteria for fibrotic myelofibrosis [3]. They also met the Italian Consensus Conference criteria for myelofibrosis which requested a diffuse bone marrow fibrosis and absence of bcr-abl translocation, plus a combination of morphologic and clinical criteria [16]. Fifty five patients were categorized under the term of prefibrotic myelofibrosis according with the 2001 WHO criteria [17] which impose absence or minimal grade of reticulin fibrosis (EUMNET grading less than 1) [18], in association with changes in bone marrow architecture, granulopoiesis hyperplasia with predominance of immature and segmented forms, and high number and clustering of atypical megakaryocytes.

The normal control population consisted in 43 student or staff members.

### Clinical and Laboratory Assessment

At the time of blood withdrawn for measurement of ECFCs, patients with myeloproliferative neoplasms were interviewed about their medical history, focusing on previous thrombotic events. In all patients, besides complete blood count and peripheral blood smear for differential count, circulating CD34+ hematopoietic progenitor cells were enumerated with standard method [5]. All patients had the mutational status for *JAK2* V617F determined using the allele specific-PCR assay on DNA purified from granulocytes, as reported [19]. A new bone marrow biopsy was obtained in the majority of the patients with primary myelofibrosis analyzed six months or later from the diagnosis.

The 13 control patients with splanchnic vein thrombosis were investigated with bone marrow biopsy, *JAK2* V617F and *MPL* mutations [20], *JAK2* exon 12 mutations [21], and clonality of hematopoiesis with the method of HUMARA in female patients [22], and myeloproliferative neoplasm was excluded.

The ECFCs assay method was that originally reported by Ingram et al [14]. In brief, a median of 90×10^6^ mononuclear cells (MNCs) (range 30–520) were plated on collagen-coated colture dishes (BD Biosciences, San Josè, CA, USA) in the presence of endothelial cell growth medium EBM-2 MV Bullet Kit (Lonza), and maintained at 37°C in 5% CO2 and humidified atmosphere. Discard of non-adherent cells and first medium change were performed after 2 days: thereafter medium was changed three times a week. The number of ECFCs-derived colonies was scored after 28 days by an inverted microscope. The outgrowth of endothelial cells from adherent MNCs was characterized by the formation of a cluster of cobblestone-appearing cells.

### Statistical analysis

ECFCs were measured in frequency per 10^7^ MNC. Within-subject variance of ECFCs assay was calculated as the mean square error from an analysis of variance model with “subject” as main effect. We found that the frequency of ECFCs had a within subject coefficient of variation of 1.2. From this measure, we estimated that the number of subjects needed to detect difference in group medians in an observation study was 43 per group [23].

The high coefficient of variation of ECFCs measurement precluded an objective definition of an individual as having increased level of ECFCs. However, we found that in repeated measures of normal subjects, the frequency of ECFCs never was greater than 0.59/10^7^ MNCs. On the basis of this consideration and for the purpose of this analysis, we used as operation definition of increased frequency of ECFCs a value greater than the upper level of normal.

In the cross sectional study, we explored the potential of using patients characteristics to identify the association between patients with increased ECFCs frequency and age, sex, disease duration, hemoglobin, white blood cell count, platelet count, number of circulating CD34+ hematopoietic progenitor cells, serum lactate dehydrogenase value, V617F mutational status of *JAK2*, diagnostic category and prognostic score (International Working Group-Myelofibrosis Research and Treatment, IWG-MRT [24]), at the time of examination. The best cut-point of hematological markers to identify individuals with increased ECFC frequency were evaluated by constructing receiver-operating characteristics (ROC) curves, which depicted the relationship between true positive (sensitivity) and false positive (1-specificity) test results. For predicting increased level of ECFCs we performed logistic regression analysis in a univariate and multivariate setting that included each characteristic and all interaction terms. We further determined the sensitivity, specificity, positive predictive value, positive likelihood value, and negative likelihood ratio for increased frequency of ECFCs in predicting the markers that were statistically significantly better performers.

Results were considered statistically significant when P values were less than 0.05. All analyses were performed using STATISTICA 9.0 software (Statsoft Inc, Tulsa, OK, USA).

## Results

**Table 1** describes the characteristics of study participants at the time of ECFC sample collection by their initial diagnosis. We limited the sample to patients who had no treatment effects. Recruitment selection resulted in high prevalence of cases with prefibrotic myelofibrosis, and in a population of patients with primary myelofibrosis in fibrotic stage mostly in the low- or intermediate 1- risk category according the IWG prognostic classification. Prefibrotic myelofibrosis patients maintained low IWG risk score in 82% of the cases, and absent or grade 1 bone marrow fibrosis in 84%.

**Table 1:** Participant Characteristics, by Diagnostic Category.

	Primary Myelofibrosis (Fibrotic Stage) (n = 106)	Prefibrotic Myelofibrosis (n = 49)	Polycythemia Vera or Essential Thrombocythemia (n = 59)*
Age, y, mean (range)	53.5 (21–83)	44.5 (22.72)	44.1 (17–86)
Male sex, male, n (%)	63 (59)	22 (40)	27 (45)
Time from diagnosis (months), mean (range)	57.1 (0–336)	60.3 (0–314)	36.2 (0–324)
Hemoglobin (g/dl), mean (range)	11.9 (5.1–18.3)	12.9 (8.8–16.7)	14.2 (11.1–18.1)
White blood cell count (×10^9^/L), mean (range)	11.6 (1.2–55)	7.8 (2.6–25.1)	10.9 (3.1–32.7)
Platelet count (×10^9^/L), mean (range)	368 (32–1186)	442 (22–909)	655 (110–1890)
Circulating CD34+ hematopoietic cells (×10^6^/L), mean (range)	84.8 (0.3–1948)	14.9 (0.4–165)	5.8 (0.6–26)
Spleen index, cm^2^, mean (range)	232 (90–1026)	151 (90–600)	114 (90–360)
Serum lactate dehydrogenase (units/L), mean (range)	903 (156–2588)	587 (214–1786)	365 (159–586)
*JAK2* V617F mutational status, n (%):wild typeheterozygoushomozygous	42 (40)37 (35)27 (25)	17 (35)28 (57)4 (8)	14 (24)30 (51)15 (25)
IWG-MRT risk score, n (%):lowintermediate-1intermediate-2high	74 (70)16 (15)12 (11)4 (4)	40 (82)5 (10)4 (8)0 (0)	NA
Bone marrow fibrosis, n (%)§:grade 0grade 1grade 2grade 3	0 (0)23 (32)24 (33)25 (35)	19 (62)7 (22)3 (10)2 (6)	NA
History of splanchnic vein thrombosis, n (%)Portal or mesenteric vein thrombosisBudd-Chiari syndrome	11 (10)10 (9)1 (1)	20 (41)16 (33)4 (8)	4 (7)3 (5)1 (2)
Mean time from SVT thrombosis to sample collection, months, (range)	97.2 (2–288)	47.5 (4–168)	24.5 (0–60)
Patients in whom SVT was diagnosed at the time of diagnosis of myeloproliferative neoplasm, n (%)	9 (82)	18 (90)	4 (100)
Patients with history of arterial thrombosis (stroke, infarction, peripheral artery disease), n (%)	3 (3)	1 (2)	0 (0)
Patients with history of deep vein thrombosis, n (%)	2 (2)	0 (0)	1 (2)

SVT = Splanchnic vein thrombosis.

* The group consisted in 38 patients with diagnosis of polycythemia vera and 21 patients with diagnosis of essential thrombocythemia.

§ Bone marrow fibrosis was graded according EUMNET criteria^18^ Bone marrow biopsy was evaluated at the time of ECFCs assay in 72 patients with a diagnosis of primary myelofibrosis and 31 with a diagnosis of prefibrotic myelofibrosis.

Normal controls consisted in 20 males (58%), and had a median age of 52 years (range 32–68). The median frequency of ECFCs was 0.05/10^7^ MNCs, with a range from 0 to 0.59/10^7^ MNCs. The frequency of ECFCs was not different between females and males and there was no correlation between ECFCs frequency and age.

Patients with prefibrotic myelofibrosis had the highest value of ECFCs frequency (median = 0.31/10^7^ MNCs), which was significantly higher than normal subjects (P = 0.04) and patients with primary myelofibrosis or polycythemia vera/essential thrombocythemia whose median value was 0 (P = 0.001 and 0.03, respectively).

Six patients (10%) with polycythemia vera or essential thrombocythemia had elevated frequency of ECFCs (greater than 0.59/10^7^ MNCs). No characteristic distinguished patients with elevated ECFCs frequency from those with non-elevated frequency. Nineteen patients (18%) with primary myelofibrosis, fibrotic stage, had elevated frequency of ECFCs, which was consistent among female patients (odds ratio = 3.09; 95% CI = 1,09–8.78; P = 0.03), those aged less than 50 (odds ratio = 3.25, 95% CI = 1.14–9.2, P = 0.02), with a history of splanchnic vein thrombosis (odds ratio = 7.56; 95% CI = 1.98–28.8; P = 0.002). Twenty-one patients (43%) with prefibrotic myelofibrosis had elevated frequency of ECFCs which was consistent among those with a history of splanchnic vein thrombosis (odds ratio = 7.42; 95% CI = 2.09–26.34; P = 0.001).

By considering the whole cohort of patients with myeloproliferative neoplasms, determination of the optimal cut-point to identify elevated ECFCs for hemoglobin, white blood cell count and platelet count yielded the following values: hemoglobin 13.7 g/dL or lower, white blood cell count 7.8×10^9^/L or lower, platelet count 400×10^9^/L or lower (**Table 2**). We used the interaction term of the three hematological parameters (hemoglobin, white blood cell count, and platelet count), and we assigned the name of “non-active disease” for those patients with the values lower than the cut-points. **Table 3** shows the results of univariate and multivariate analysis. The association with elevated frequency of ECFCs was modest in the patients who were females, younger than 50 years, or with the diagnosis of prefibrotic myelofibrosis, but was greater and highly statistically significant in patients who had a non-active disease, and was greatest in those who had a history of splanchnic vein thrombosis. At multivariate analysis the history of spanchnic vein thrombosis and the phenotype of non-active disease resulted independent predictors of increased ECFCs frequency.

**Table 2:** Results of Univariate and Multivariate Analysis of Disease Characteristics Associated with Increased Frequency of ECFCs.

Variable	Patients, n/n	Patients with Variable when Increased ECFCs Frequency is Present, n	Univariate analysis		Multivariate analysis	
			Unadjusted Odds Ratio for Increased ECFCs Frequency (95% CI)	P value	Adjusted Odds Ratio for Increased ECFCs Frequency (95% CI)	P value
Female sex	102/214	29	2.24 (1.09–4.69)	0.026		
Age lower than 50 years	99/214	17	2.72 (1.28–5.68)	0.008		
Diagnosis of prefibrotic myelofibrosis	56/214	21	2.84 (1.36–5.81)	0.005		
Phenotype of non-active disease	27/214	13	4.67 (1.81–11.94)	0.001	4.43 (1.45–13.49)	0.008
History of splanchnic vein thrombosis	35/214	20	7.63 (3.34–17.91)	<0.001	6.61 (2.54–17.16)	<0.001

ECFCs = Endothelial colony forming cells.

**Table 3:** Ability to Predict the History of Splanchnic Vein Thrombosis, and the Phenotype of Non-Active Disease by Increased Frequency of ECFCs.

Marker			Sensitivity (95% CI), %	Specificity (95% CI)%	Positive LR (95% CI)	Negative LR (95% CI)	Positive Predictive Value,%
	History of splanchnic vein thrombosis, n	No history of splanchnic vein thrombosis, n					
ECFCs>0.59/10^7^ MNCs	20	25	57.1 (40.0–73.7)	86.4 (80.6–91.0)	4.2 (2.6–6.7)	0.5 (0.3–0.7)	44.4 (29.6–60.0)
ECFCs<0.59/10^7^ MNCs	15	159					
	Phenotype of non-active disease	Phenotype of active disease					
ECFCs>0.59/10^7^ MNCs	13	33	48.1 (28.7–68.0)	80.8 (74.1–86.4)	2.5 (1.5–4.1)	0.6 (0.4–0.9)	28.2 (16.0–43.4)
ECFCs<0.59/10^7^ MNCs	14	139					

LR = likelihood ratio.

**Table 4** shows the prediction ability of increased frequency of ECFCs to identify the phenotype of non-active disease, and history of splanchnic vein thrombosis. The greatest positive likelihood ratio of increased ECFC was for history of splanchnic vein thrombosis, that indicates that the odds of having a history of splancnic vein thrombosis increased by 4.2 times if the test result was positive as compared with a test negative. History of splanchnic vein thrombosis had also the greatest predictive value (44%).

**Table 4:** Sensitivity, Specificity, and Cut-off Value for the Ability of Hematological Parameters to Identify Patients with Increased Frequency of ECFCs.

Parameter	Sensitivity %	Specificity %	Cut-off value	AUC (95% CI)	P level
Hemoglobin, g/dL	85	36	≤13.8	0.59 (0.52–0.66)	0.037
White blood cell count, 10^9^/L	70	61	≤7.8	0.67 (0.61–0.74)	<0.001
Platelet count, 10^9^/L	66	53	≤400	0.57 (0.49–0.64)	0.045

AUC = Area under the curve.

To evaluate the biological effects of splanchnic vein thrombosis on frequency of ECFCs, we studied 5 patients with Budd Chiari syndrome and 8 with portal vein thrombosis without an underlying myeloproliferative disease. They were 3 males (23%), and their mean age was 52.8 years (range 36–67). The time from diagnosis of splanchnic vein thrombosis to ECFCs assay ranged from 0 to 96 months, mean 24 months, i.e. shorter than in patients with prefibrotic myelofibrosis or fibrotic type primary myelofibrosis. Overall, 10 (77%) of patients had at least one thrombophilia abnormality or one condition or disease known to predispose to thrombosis. Five patients were taking oral contraceptives, and one was on hormone replacement therapy. The median frequency of ECFCs was significantly lower than in patients with myeloproliferative neoplasm-associated splanchnic vein thrombosis of our cohort (median, 0/10^7^ MNCs vs. 1/10^7^ MNCs; P = 0.01), and no patient had elevated ECFC frequency.

## Discussion

Our analysis of a large cohort of patients with Ph-neg myeloproliferative neoplasms shows that mobilization of endothelial progenitor cells is consistently higher in patients who received a diagnosis of prefibrotic myelofibrosis. However, the association between elevated ECFCs frequency and the diagnosis of prefibrotic myelofibrosis did not persist after adjustment for the contribution of the other potential influential factors, like age less than 50 years, female sex, a phenotype of non-active disease, and history of splanchnic vein thrombosis. As a matter of fact, the phenotype of non-active disease, and history of splanchnic vein thrombosis resulted independently associated with high frequency of circulating ECFCs at multivariate analysis. The definition of active and non-active disease originated by this data set assumes absence of active myeloproliferation. A patient with non-active disease is one with normal or decreased hemoglobin (less than 13.7 g/dL), leukocyte count (less than 7.8×10^9^/L), and platelet count (less than 400×10^9^/L). This definition is thus based on fewer and simpler parameters than those defining the low-risk category of patients with primary myelofibrosis according to the IWG-MRT criteria that allow higher number of white blood cells (25×10^9^/L) and no cut-off for platelet count.

Despite the significant association of increased ECFCs frequency with history of splanchnic vein thrombosis and the phenotype of non-active disease, the ability to use ECFCs elevation as a biomarker for the prediction of these characteristics was moderate, being the predictive value 44.4% and 28.2%, respectively. This could be justified by the relatively low prevalence of history of splanchnic vein thrombosis and non-active phenotype in our cohort, considering that the predictive ability of any biomarker depends on the prevalence of the predicted event in that population. So, in populations with a larger proportion of cases with splanchnic vein thrombosis and non-active disease, higher predictive ability is expected. Furthermore, the 1.2 coefficient of variation of ECFCs assay we have determined in the preliminary analysis of this study, implies a high proportion of false-negative and false-positive patients in the ascertainment of high mobilizers.

The association between splanchnic vein thrombosis and elevated frequency of ECFCs generates the question whether the thrombotic episode per se mobilizes ECFCs. For this question, we intentionally selected a cohort of patients with splanchnic vein thrombosis non associated with a myeloproliferative neoplasm. We documented that the association between increased ECFCs frequency and splanchnic vein thrombosis was lacking. So, with inductive reasoning we concluded that the association depends on the underlying myeloproliferative neoplasm that predisposes patients both to develop thrombosis and to mobilize ECFCs. This result suggests several important future directions for research addressing the role of ECFCs on the splanchnic thrombotic event.

In the interpretation of the results of this study we set off the biological strength and the clinical relevance of the correspondence between high ECFCs frequency and non-active phenotype and high risk of splanchnic vein thrombosis, respectively. Accordingly, we interpreted high mobilization of ECFCs in the realm of myeloproliferative neoplasms as the biological hallmark of a separate category of patients, more than of a phase in the evolution of the myeloproliferative process. This is also supported by the fact that high ECFCs mobilizers were mostly females and had preferentially young age.

The idea that patients with non-active phenotype and high-risk of splanchnic vein thrombosis would identify a separate category of patients is not fully original. Indeed, patients we have identified through the measurement of mobilized endothelial progenitor cells are similar to those we singled out by pattern recognition in 1991 and we defined with the name of “atypical myeloproliferative disorder with high thrombotic risk and slow disease progression” [25]. Moreover, the patients' characteristics identified in this work convincingly accommodate the series of cases with an occult or latent myeloproliferative disorder associated with splanchnic vein thrombosis in which an indolent, atypical or unapparent myeloproliferative process is associated with the thrombotic event [26]–[30].

The major limit of our experimental approach is that the appreciation of high endothelial progenitor cell frequency was obtained with an assay that has inherent performance limitations due to low analytical read out, repeatability, and practicality. Thus, the importance of this study relies on the appreciation that a biomarker is able to identify a separate category of myeloproliferative neoplasm. However, there is an urgent need to arrive either to an acceptable standardization of ECFCs assessment, or to find more feasible, highly correlated, biomarkers that would be clinically included in the work-up of early stages, indolent or unclassifiable myeloproliferative neoplasms. The recognition of such a category of patients can help in unraveling the phenotypic mimicry of myeloproliferative neoplasms and in a more homogenous case reporting in clinical studies. Moreover, since splanchnic vein thrombosis may occur without the signs of an underlying myeloproliferative neoplasm, having a new diagnostic biomarker could help health care professionals be more successful in bringing about the diagnosis of the associated myeloproliferative neoplasm.
